# 

**Published:** 1909-08

**Authors:** 


Sixty-fifth Year.

VOL. LXV. AUGUST, 1909. No. 1

### BUFFALO MEDICAL JOURNAL

Established 1845 by AUSTIN FLINT M. D.

LIBRARY SURGEON GENERAL'S OFFICE AUG 1 - 1909

LIBRARY Indexed S. G. O.

EDITOR:
WILLIAM WARREN POTTER, M. D.

ASSISTANT EDITORS:
WILLIAM C. KRAUSS, M. D. NELSON W. WILSON, M. D.

ASSOCIATE EDITORS:
JAMES WRIGHT PUTNAM, M. D. ERNEST WENDE, M.D.
JOHN PARMENTER, M. D. JOHN A. MILLER, PH. D.
MAUD J. FRYE, M. D.

